# Clinical features and characteristics of uveitis associated with juvenile idiopathic arthritis in Japan: first report of the pediatric rheumatology association of Japan (PRAJ)

**DOI:** 10.1186/s12969-019-0318-5

**Published:** 2019-04-11

**Authors:** Junko Yasumura, Masato Yashiro, Nami Okamoto, Kosuke Shabana, Hiroaki Umebayashi, Naomi Iwata, Yuka Okura, Tomohiro Kubota, Masaki Shimizu, Minako Tomiita, Yasuo Nakagishi, Kenichi Nishimura, Ryoki Hara, Mao Mizuta, Takahiro Yasumi, Fumiya Yamaide, Hiroyuki Wakiguchi, Masao Kobayashi, Masaaki Mori

**Affiliations:** 1 Department of Pediatrics, Hiroshima University Graduate School of Biomedical and Health Sciences, 1-2-3 Kasumi, Minami-ku, Hiroshima, 734-8551 Japan; 2 Department of Pediatrics, Okayama University Hospital, 2-5-1 Shikata-cho, Kita-ku, Okayama, 700-8558 Japan; 3 Department of Pediatrics, Osaka Medical College, 2-7 Daigaku-machi, Takatsuki, 569-8686 Japan; 4 Department of General Pediatrics, Miyagi Children’s Hospital, 4-3-17 Ochiai, Aoba-ku, Sendai, 989-3126 Japan; 5 Department of Immunology and Infectious Diseases, Aichi Children’s Health and Medical Center, 7-426 Morioka-cho, Obu, Aichi, 474-8710 Japan; 6 Department of Pediatrics, KKR Sapporo Medical Center, 6-3-40 Hiragishi 1-jo, Toyohira-ku, Sapporo, 062-0931 Japan; 7 Department of Pediatrics, Kagoshima University Hospital, 8-35-1 Sakuragaoka, Kagoshima, 890-0075 Japan; 8 Department of Pediatrics, Graduate School of Medical Sciences, Kanazawa University, 13-1 Takara-machi, Kanazawa, 920-8641 Japan; 9 Department of Allergy and Rheumatology, Chiba Children’s Hospital, 579-1 Heta-cho, Midori-ku, Chiba, 266-0007 Japan; 10 Department of Pediatric Rheumatology, Hyogo Prefectural Kobe Children’s Hospital, 1-6-7 Minatojimaminami-machi, Chuo-ku, Kobe, 650-0047 Japan; 11 Department of Pediatrics, Yokohama City University Graduate School of Medicine, 3-9 Fukuura, Kanazawa-ku, Yokohama, 236-0004 Japan; 12 Department of Pediatrics, Kyoto University Graduate School of Medicine, 54 Shogoin Kawahara-cho, Sakyo-ku, Kyoto, 606-8507 Japan; 13 Department of Pediatrics, Chiba University Graduate School of Medicine, 1-8-1 Inohana, Chuo-ku, Chiba, 260-8670 Japan; 14 Department of Pediatrics, Yamaguchi University Graduate School of Medicine, 1-1-1 Minamikogushi, Ube, 755-8505 Japan; 15 Department of Lifetime Clinical Immunology, Graduate School of Medical and Dental Sciences, Tokyo Medical and Dental University, 1-5-45 Yushima, Bunkyo-ku, Tokyo, 113-8510 Japan

**Keywords:** Juvenile idiopathic arthritis, Uveitis, Epidemiology, Asian

## Abstract

**Background:**

Although there are many reports on Juvenile Idiopathic arthritis-associated uveitis (JIA-U) from various countries, especially from Europe and North America, there are few reports from Asia. Our aim was to investigate the epidemiology, characteristics and predictors of JIA-U in Japan.

**Methods:**

Data were retrospectively collected on 726 patients with JIA from medical records as of April 2016 at 15 medical centers specialized in pediatric rheumatic diseases. Of these, patients with uveitis were further investigated for the specific characteristics of this manifestation.

**Results:**

The prevalence of uveitis was 6.1% in the 726 JIA patients examined. Incidence of uveitis was significantly higher in patients with an earlier arthritis onset (2.6-vs.-5.8 years, *P* < 0.0001), oligoarthritis (16.1%-vs.-1.6%, *P* < 0.001), or anti-nuclear antibodies. On the contrary, it was significantly less common in patients with rheumatoid factor or anti-cyclic citrullinated peptide antibodies. A history of using methotrexate (MTX), infliximab or adalimumab was also associated with uveitis occurrence. The median age at uveitis diagnosis was 5 years, and the median time from arthritis onset to uveitis diagnosis was 2 years. The occurrence of anterior and bilateral uveitis was 79.3 and 53.7%, respectively. There were no symptoms at uveitis diagnosis in 58.5% of cases. Complications arising between the time of uveitis diagnosis and the last observation increased from 31.7 to 56.1%; in particular, cataract was increased 3-fold. While no patients lost their vision, 61.9% did not recover normal vision (≥ 1.0), and in many cases active uveitis persisted, especially in males. In addition to steroid eye drops (97.6%) and MTX (15.4%), biological agents were used for treating the uveitis in 41.5% of patients.

**Conclusions:**

The epidemiology, characteristics and predictors of JIA-U in Japan are described here for the first time. Although the prevalence of JIA-U in Japan is lower than in predominantly Caucasian cohorts, as reported from North America and Europe, the epidemiology, characteristics and predictors were found to be similar.

## Background

Uveitis is the most common extra-articular manifestation of juvenile idiopathic arthritis (JIA); it is a serious manifestation carrying the risk of blindness if treatment is delayed or inadequate. The prevalence of JIA-associated uveitis (JIA-U) has been reported as ranging from 4.7 to 20.5% [1–6] with local differences noted. Numerous reports have identified risk factors for JIA-U such as female sex, oligoarthritis, earlier arthritis onset, ANA-positivity, and RF-negativity in predominantly Caucasian cohorts [2, 7, 8]. Do these characteristics also apply to JIA-U in East Asia? To date, there are very few reports from East Asia and the epidemiology, characteristics and risk factors for JIA-U in Japan are unclear.

Although we, the members of the Pediatric Rheumatology Association of Japan (PRAJ), issued recommendations for ophthalmologic screening intervals for JIA patients in Japan [9, 10] (Table 1), these were based on other countries’ guidelines [2, 11]. In any event, these recommendations were issued only one year before this study of patients in Japan, and therefore such recommendations did not apply to most of the patients included here. Given that the prevalence and characteristics of JIA-U in Japan were unclear, and pediatricians and ophthalmologists had little knowledge of the management of JIA-U, investigating these issues specifically in Japanese patients was warranted. Accordingly, in the present study, we aimed to establish the epidemiology, clinical characteristics, and risk factors for JIA-U in Japan and to compare these with data reported from other countries. Thus, we reviewed the charts of outpatients making regular hospital visits as of April 2016.

**Table 1:** Recommendations for ophthalmologic screening intervals for JIA patients in Japan [9, 10]

JIA category	ANA (titer)^a^	Ophthalmologic screening intervals	
		Onset age of ≤6 years old	Onset age ≥ 7 years old
・≤4 years from onset of arthritis			
Oligoarthrits, RF-negative polyarthritis, undifferentiated arthritis	≥160	Every 3 months	Every 6 months
	< 160	Every 6 months	Every 6 months
Psoriatic arthritis whose onset age is <4 years old	Regardless	Every 3 months	–
Others	Regardless	Every 12 months	Every 12 months
・4 < years, ≤7 years from onset of arthritis			
Oligoarthrits, RF-negative polyarthritis, undifferentiated arthritis	≥160	Every 6 months	Every 12 months
	< 160	Every 12 months	Every 12 months
Psoriatic arthritis whose onset age is <4 years old	Regardless	Every 6 months	–
Others	Regardless	Every 12 months	Every 12 months
・>7 years from onset of arthritis: Every 12 months.			

*JIA* juvenile idiopathic arthritis, *ANA* antinuclear antibody, *RF* rheumatoid factor, ^a^fluorescent antibody testing

## Methods

### Study setting

This is a retrospective study, approved by the Ethics Review Board of Tokyo Medical and Dental University (No. M2015–537), the main study center, on March 4th, 2016, after which approval was obtained from each of the 15 other participating medical centers. Guardians and patients were provided information by means of an opt-out form.

### Study design and patients

First, we sent questionnaires to pediatricians who are members of the PRAJ belonging to 15 medical centers to gather data on the characteristics of JIA in Japan. We analyzed data from outpatients with JIA who had regularly visited the hospital as of April 2016. All patients were classified according to the International League of Associations for Rheumatology (ILAR) criteria [12]. Second, we undertook a further questionnaire survey for patients identified as having uveitis in the first questionnaire, and gathered more detailed information about uveitis features from their ophthalmology charts using this second questionnaire. Patients who had already discontinued follow-up of JIA as of April 2016 were not included.

### Data collection

All patient data including ophthalmic records were collected from medical histories and were recorded by pediatricians. The following parameters were evaluated for the primary investigation: sex, age as of April 2016, duration of disease, age at arthritis diagnosis, type of JIA classified by ILAR criteria, laboratory data including anti-nuclear antibody (ANA), rheumatoid factor (RF), anti-cyclic citrullinated peptide (CCP) antibody, serum matrix metalloproteinase-3 (MMP-3), and drugs used for treatment. An ANA titer of ≥1:160 by fluorescent antibody testing was designated positive. Data on ANA, RF and MMP-3 were acquired at the time of arthritis diagnosis. The following parameters were evaluated in the second investigation: race of parents, family history of uveitis, infection history, possession of HLA-B27, location of uveitis, age at uveitis diagnosis, time from arthritis onset to uveitis diagnosis, timing of uveitis diagnosis, eye manifestations at uveitis diagnosis, eye complications due to uveitis at the first and last observation, activity of arthritis at uveitis diagnosis, current activity of arthritis, current activity of uveitis, visual acuity at uveitis diagnosis and at present, drugs used for treatment. Visual acuity was recorded as a decimal unit using the Landolt ring method where 1.0 refers to 20/20 of the Snellen fraction, whereas 0.1 refers to 20/200 [13].

### Statistical analysis

Statistical analysis was performed using JMP pro® Version 13 software (SAS Institute Inc., Cary, NC, USA). The Wilcoxon rank sum test or Welch’s t test were used for comparison of continuous variables between two groups, and categorical variables were analyzed using the Chi-Square test or Fisher’s exact test (two-sided). Odds ratios (ORs) are presented with 95% confidence intervals (CI). The time from onset of arthritis to diagnosis of uveitis was analyzed by the Kaplan-Meier method. A *p*-value < 0.05 was considered significant.

## Results

### Comparison of characteristics of JIA patients with and without uveitis

We collected data from 730 patients with JIA at 15 medical centers. Four patients were excluded due to many missing data. Of the remaining 726 JIA patients, 44 (6.1%) had uveitis. Table 2 shows the characteristics of patients with and without uveitis. There were no significant differences between the two groups in sex or age at the last visit. Patients with uveitis had been significantly younger at arthritis onset (2.6-vs.-5.8 years, *P* < 0.0001) and the duration of disease was significantly longer (9.1-vs.-5.1 years, *P* < 0.0001) than in patients without uveitis. The most frequent disease type in patients with uveitis was oligoarthritis (81.8%), followed by RF-negative polyarthritis, but there were no patients with systemic arthritis or RF-positive polyarthritis. There were very few patients with psoriatic arthritis or non-classified arthritis in either group. The rate of uveitis in oligoarthritis patients was 16.1% and in all other JIA patients 1.6%; thus, the risk of uveitis in oligoarthritis was 10-fold higher (Table 6). In addition, the frequency of ANA-positive patients was significantly higher in the group with uveitis (52.3%-vs.-22.2%, *P* < 0.0001), with a tendency towards higher ANA titers than patients without uveitis (ANA titers ≥1:640; *P* = 0.018) (data not shown). The frequency of RF-positive cases (2.4%-vs.-24.5%, *P* = 0.0004) and those with anti-CCP antibodies (0%-vs.-26.2%, *P* < 0.0001) was significantly lower in the uveitis group. No significant differences in serum MMP-3 levels at JIA diagnosis were noted between the two groups. The proportion of patients with a history of using oral Methotrexate (MTX) was significantly higher in patients with uveitis (95.5%-vs.-77.0%, *P* = 0.0022). Similarly, significant differences were noted for infliximab (IFX), adalimumab (ADA), and tocilizumab (TCZ) use: IFX and ADA were more frequently used in the uveitis group, whereas TCZ was more frequently used by patients without uveitis.

**Table 2:** Comparison of characteristics in 682 non-uveitis patients vs. 44 JIA-U patients

	JIA total	without uveitis	with uveitis	*P* - value^c^
Number of Patients, N (%)	726	682 (93.9%)	44 (6.1%)	
Variables				
Female, N (%)	492 (67.8%)	461 (67.6%)	31 (70.5%)	0.743
Age at the last visit, N	724	680	44	
median ^a^ (yrs.)	12.9 (8.9–17.2)	12.8 (8.8–17.2)	13.6 (10.1–18.3)	0.191
Duration of disease at the last visit, N	718	674	44	
median ^a^ (yrs.)	5.4 (2.8–9.2)	5.1 (2.7–8.8)	9.1 (6.5–14.3)	< 0.0001^*^
Age at arthritis onset, N	720	676	44	
median ^a^ (yrs.)	5.5 (2.7–10.3)	5.8 (2.8–10.4)	2.6 (1.6–5.1)	< 0.0001^*^
Subtypes of JIA				
Oligo persistent, N (%)	184 (25.3%)	155 (22.7%)	29 (65.9%)	< 0.001*
Oligo extended	40 (5.5%)	33 (4.8%)	7 (15.9%)	0.0076*
Poly RF (−)	95 (13.1%)	90 (13.2%)	5 (11.4%)	1.000
Poly RF (+)	152 (20.9%)	152 (22.3%)	0 (0%)	< 0.001*
Systemic	204 (28.1%)	204 (29.9%)	0 (0%)	< 0.001*
Psoriatic	4 (0.6%)	4 (0.6%)	0 (0%)	1.000
Enthesitis related	37 (5.1%)	35 (5.1%)	2 (4.5%)	1.000
Undifferentiated	10 (1.4%)	9 (1.3%)	1 (2.3%)	0.467
Blood test				
ANA tested	615	571	44	
Positive^b^, N(%)	150 (24.4%)	127 (22.2%)	23 (52.3%)	< 0.0001*
RF tested	592	551	41	
Positive, N (%)	136 (23.0%)	135 (24.5%)	1 (2.4%)	0.0004*
Anti-CCP antibody tested	445	404	41	
Positive, N (%)	106 (23.8%)	106 (26.2%)	0 (0%)	< 0.0001*
MMP-3 tested	552	521	31	
MMP-3 (ng/ml), mean ± SD	169.0 ± 301.3	170.6 ± 308.7	142.0 ± 123.4	0.27
Medication Use				
Methotrexate, N (%)	567 (78.1%)	525 (77.0%)	42 (95.5%)	0.0022*
Infliximab	30 (4.1%)	18 (2.6%)	12 (27.3%)	< 0.0001*
Etanercept	81 (11.2%)	78 (11.4%)	3 (6.8%)	0.4625
Adalimumab	124 (17.1%)	109 (16.0%)	15 (34.1%)	0.0058*
Tocilizumab	297 (40.9%)	289 (42.4%)	8 (18.2%)	0.0014*
Abatacept	24 (3.3%)	24 (3.5%)	0 (0%)	0.3904*

^a^interquartile ranges: 25th – 75th percentile, ^b^fluorescent ANA ≥1:160, ^c^Statistically significant at 5% level of significance; *p = < 0.05

Chi-square tests, Fisher’s extract test (two-tailed test), Wilcoxon rank sum test

### Clinical characteristics of patients with uveitis

We obtained data for a further analysis of 41 of the 44 patients with uveitis. Three patients were excluded due to lack of data from their physicians. Of the 41 patients, 38 had Japanese parents, one had Chinese parents, one had a Chinese and a Japanese parent, and the other had a Chinese and an Iranian parent. They were found to have no family history of uveitis or history of preceding infections. HLA-B27 had been tested only in 34.1% (14 of 41 patients) because of not being covered by insurance. Two patients were positive, and 12 patients were negative.

We were able to obtain data on the site of uveitis from only 29 of the 41 patients because of poor record keeping by ophthalmologists in some centers. Uveitis in 29 patients was anterior in 79.3% (*N* = 23), panuveitis in 13.8% (*N* = 4), posterior in 3.5% (*N* = 1), and both anterior and posterior in 3.5% (N = 1). Uveitis was bilateral in 53.7% of these 41 patients.

**Fig. 1:**
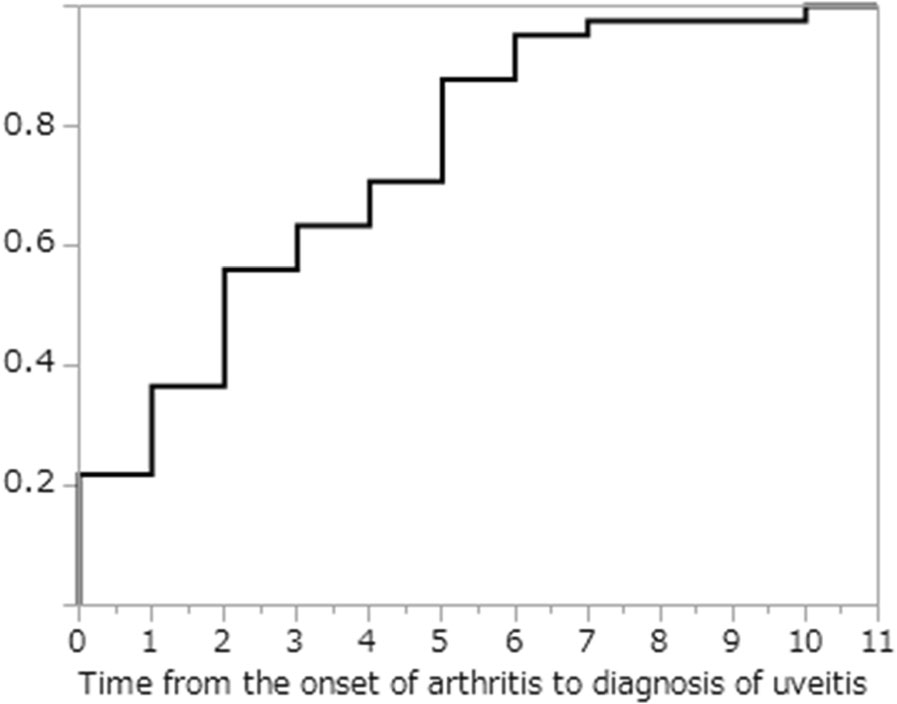
Cumulative uveitis onset rate (vertical axis) in 41 JIA-U patients, including 2 children developing uveitis before onset of arthritis (time 0). The horizontal axis indicates years from the onset of arthritis to diagnosis of uveitis

The median age at uveitis diagnosis was 5 years (interquartile ranges: 25th–75th percentile, 3–7); uveitis occurred before the 5th and 8th birthday in 43.9 and 80.5% of patients, respectively. Median time from arthritis onset to uveitis diagnosis was 2 years (interquartile ranges: 25th–75th percentile, 1–5). Time from arthritis onset to uveitis diagnosis is shown Fig. 1, including two patients in which uveitis was diagnosed even before the arthritis. Time from arthritis onset to uveitis diagnosis was < 4 years in 63.4% and < 7 years in 95.1% of patients. The timing of uveitis diagnosis was “prior to arthritis onset” in 4.9%, “at the same time as arthritis diagnosis” in 36.6%, “during on medication” in 36.6%, and “during off medication remission” in 19.5% of the patients. Of the 15 (36.6%) patients who developed uveitis during arthritis treatment, 13 were being treated with oral MTX, one with non-steroidal anti-inflammatory drugs(NSAIDs), and the remaining patient with NSAIDs plus glucocorticoids (GC). One patient used a biological agent, TCZ. Uveitis developed in 9 patients from 4 months to 6 years after discontinuation of arthritis treatment; for 8 of the 9 it was within 3 years. While 56.1% had arthritis at uveitis diagnosis, 41.5% of patients exhibited only persistent uveitis at the final observation, and not arthritis.

Ocular symptoms were present at uveitis diagnosis in 36.6% of patients, whereby ocular hyperemia accounted for 46.7% and decreased vision accounted for 46.7% (Table 3). Ocular complications were noted in 31.7% of patients at diagnosis (three were unknown) and in 56.1% at the final observation. The major complications at diagnosis were posterior synechia of the iris (46.2%), followed by cataract (30.8%), band-keratopathy (30.8%), and glaucoma (23.1%). At the time of the last observation, the number of patients with cataract had increased 3-fold since uveitis diagnosis (Table 4). There were no significant differences in oral GC use between patients with or without cataracts at the final observation. There were also no significant differences between patients with or without complications at diagnosis in the proportions of patients with active uveitis at the final observation (*P* = 0.09, OR: 3.6, 95% CI: 0.9–14.4). However, patients with complications at diagnosis had significantly more complications at the final observation (*P* = 0.014, OR: 5.57, 95% CI: 1.51–20.51). No significant differences were noted between the presence or absence of complications at uveitis diagnosis and the age of the patients at the time of uveitis diagnosis, or time from arthritis onset to uveitis diagnosis.

**Table 3:** Ocular symptoms at uveitis diagnosis (*N* = 41^a^)

	N (%)
Ocular symptom (+)	15 (36.6%)
Decreased vision	7 (46.7%)
Ocular hyperemia	7 (46.7%)
Photophobia	2 (13.3%)
Ocular pain	1 (6.7%)
Blurred vision	1 (6.7%)
Myodesopsia (floaters)	1 (6.7%)
White pupil	1 (6.7%)

^a^2 data were unknown. There were patients who had multiple symptoms

**Table 4:** Ocular complications in JIA-U patients^a^

	at uveitis diagnosis (*N* = 41^b^) N (%)	at the last visit (*N* = 41) N (%)
Ocular complication (+)	13 (31.7%)	23 (56.1%)
Cataract	4 (30.8%)	12 (52.2%)
Glaucoma	3 (23.1%)	5 (21.7%)
Posterior synechia	6 (46.2%)	4 (17.4%)
Band-keratopathy	4 (30.8%)	7 (30.4%)
Other complications	1 (7.7%) ^c^	2 (8.7%) ^d^

^a^There were patients who had multiple complications. ^b^3 data were unknown. ^c^papilledema. ^d^divergent strabismus and Iridocyclitis scar

We obtained visual acuity data on 63 affected eyes (30 right eyes and 33 left eyes) in 39 patients. Two were excluded because of lack of data of the side of uveitis and visual acuity. We did not identify any patient who lost vision during the observation period. However, visual acuity of the affected eye less than 0.1 was noted in 6.7% at diagnosis (4 of 60 eyes: 3 data points were missing), and in 6.4% at the final observation (4 of 63 eyes). Visual acuity less than 1.0 at the final observation was noted in 61.9% (39 of 63 eyes) and less than 0.5 was noted in 22.2% (14 of 63 eyes). No significant differences were seen between complications at diagnosis and at the final observation, and prognosis of visual capacity.

### Uveitis treatment

Steroid eye drops were mainly used for uveitis treatment (97.6%). Only two patients received steroid injections into the eye. MTX was used in 34.1% (14/41 patients) at diagnosis of uveitis, and in 95.1% (39/41 patients) after diagnosis. Physicians used MTX for treating the uveitis in only 15.4% (6/39 patients) after diagnosis of uveitis. On the other hand, biological agents were used in 70.7% of patients and for treating uveitis in 41.5%. IFX (29.3%) and ADA (36.6%) were the most frequently-used agents. A history of some type of surgery was noted in 36.6% of the patients. Of these, 86.7% (13/15 patients) underwent cataract surgery, most of which were phacoemulsification and intraocular lens implantation. There was no significant association between the presence of cataract at the time of final observation and a history of surgery (*P* = 0.0834). Other surgery included vitrectomy, trabeculectomy, perioperative surgery and phototherapeutic keratectomy.

### Sex differences

Risk factors were compared between males and females in the group with uveitis (Table 5). No significant sex differences were seen for age at uveitis diagnosis. Although there was also no significant difference in age at onset of arthritis in either males or females without uveitis (6.9 ± 4.4-vs.-6.6 ± 4.3 years, *P* = 0.37, data not shown), females in the uveitis group had a significantly earlier arthritis onset (2.3 ± 1.9 vs. 4.5 ± 3.0 years old, *P* = 0.032). While the percentage of ANA-positive females at diagnosis of JIA was significantly higher in patients without uveitis (27.8%-vs.-10.1%, *P* < 0.0001, data not shown), there were no significant sex differences in the uveitis group. There was a tendency for more female than male patients to have anterior uveitis at diagnosis, but this difference was not significant (94.1%-vs.-58.3%, *P* = 0.0563). The proportion of patients without ocular symptoms at diagnosis was higher in females than males (74.1%-vs.-33.3%, *P* = 0.031). Although there was no significant sex difference in the proportion of patients with any type of complication, the proportion of males with persistent active uveitis at the final observation was significantly higher than in females (76.9%-vs.-28.6%, *P* = 0.006). In addition, there were no significant sex differences in visual acuity or decreased visual acuity at diagnosis or at final observation. Furthermore, no significant sex difference was observed in time from arthritis onset to uveitis diagnosis.

**Table 5:** Sex differences of characteristics in JIA-U patients

Variables	N	Male	N	Female	P	OR (95%CI)
mean age as of April 2016	13	11.5 ± 4.7	28	13.8 ± 6.4	0.3737	
mean age at uveitis diagnosis	13	6.5 ± 3.0	28	5.2 ± 2.8	0.2207	
mean age at arthritis onset	13	4.5 ± 3.0	28	2.3 ± 1.9	0.0324	
mean serum MMP-3 (ng/ml) at JIA diagnosis	11	145.9 ± 150.8	20	139.8 ± 109.8	0.69	
ANA ≥1:160	13	5 (38.5%)	31	17 (54.8%)	0.5098	0.51(0.14~1.93)
bilateral uveitis at uveitis diagnosis	13	6 (46.2%)	26	15 (57.7%)	0.52	0.63(0.16~2.4)
anterior uveitis at uveitis diagnosis	12	7 (58.3%)	17	16 (94.1%)	0.0563	0.09(0.01~0.89)
without eye symptoms at uveitis diagnosis	12	4 (33.3%)	27	20 (74.1%)	0.0306	5.71(1.3~25.0)
with complications at uveitis diagnosis	12	6 (50.0%)	26	8 (30.8%)	0.296	2.25(0.55~9.17)
with complications at the last visit	13	9 (69.2%)	28	13 (46.4%)	0.2	2.6(0.65~10.5)
with persistent uveitis activity at the last visit	13	10 (76.9%)	28	8 (28.6%)	0.0063	8.33(1.8~38.4)

Chi-square tests, Fisher’s extract test (two-tailed test), Welch’s t test

## Discussion

This is the first report surveying the epidemiology and characteristics of JIA-U in Japan. Our results are compared with previous reports in Table 6. It is apparent that the prevalence of JIA-U in Japan is lower than reported earlier from North America and Europe. These previous reports revealed regional differences in the prevalence of JIA-U: 11.6–20.5% in North America and Europe [1–5] and 4.7% in Taiwan [6]. Another study reported that the prevalence of JIA-U in Asia is lower than in Scandinavia as well as the United States [14]. Although these differences may be due to differences in study design, there may well also be an influence of race. Because almost all of the patients assessed in our survey were Asians, this population may have a lower incidence than predominantly Caucasian populations.

**Table 6:** Comparison of epidemiology and characteristics of JIA-U in our results vs. in previous reports

study	our study	Saurenmann, 2007 [1]	Heiligenhaus, 2007 [2]	Nordal, 2017 [5]	Angeles-Han, 2015 [18]
country	Japan	Switzerland	Germany	Norway	United States of America
prevalence	6.1%	13.1%	12%	20.5%	18%
JIA subtype					
oligo	81.8%	48%	41%	46.1%	78.9%
RF + poly	0%	0%	0%	0%	0%
Systemic	0%	0.6%	0%	0%	0%
Rate of uveitis in oligo	16.1%	20.9%	17.6%	19.6%	30.8%
Rate of uveitis in all other type	1.6%	8.3%	6.7%	21.3%	7.1%
Risk ratio of uveitis in oligo	10	2.5	2.6	0.9	4.3
female^a^	67.6% vs. 70.5%, *p* = 0.743	79.6% vs. 63.7%, *p* = 0.0009^b^	74% vs. 62%, *p* = 0.012	65.6% vs. 66.3%	76.9% vs. 70.2%, *p* = 0.332
ANA-positive (%)^a^	52.3% vs. 22.2%, *p* < 0.0001	80.9% vs. 51%, *p* < 0.0001	86% vs. 42%, *p* < 0.01	42.5% vs. 23.2%	54.9% vs. 35.9%, *p* = 0.017
RF-positive (%)^a^	2.4% vs. 24.5%, *p* = 0.0004	–	–	1.1% vs. 2.7%	0% vs. 11.1%, *p* = 0.013
the mean age of uveitis onset (yrs.)	5.6	6.2	5.2	10.8(acute) and 3.2(chronic)	median 4.8
the mean age of arthritis onset (yrs.)^a^	3.8 vs. 6.8	4.3 vs. 7.3	3.8 vs. 7.0	–	median 2.8 vs. 7.7
time from arthritis onset to uveitis diagnosis	median 2 yrs.	mean 1.8 yrs.	median 5.5 months	–	–
uveitis diagnosis before the arthritis diagnosis	4.9%	12.7%	10%	–	24%
anterior uveitis	79.3%	100%	83%	–	80%
bilateral uveitis	53.7%	60.6%	70%	–	72%
asymptomatic uveitis	58.5%	69.7%	–	–	–

^a^Comparison of patients with uveitis vs. without uveitis, *p*-value. ^b^Only girls with oligoarthritis have a risk of uveitis

Our study confirmed that oligoarthritis, earlier arthritis onset, ANA-positivity, RF-negativity and anti-CCP antibody-negativity could be risk factors for JIA-U in Japanese as well as in predominantly Caucasian populations. Several reports showed that oligoarthritis was the most common subtype of JIA with uveitis [2, 15, 16]. Likewise, in our study most patients (81.8%) with uveitis had oligoarthritis. While oligoarthritis accounts for approximately 50% of all JIA patients in North America and Europe [2, 4, 5, 17], the proportion is smaller in Japan and many patients have systemic and RF-positive polyarthritis. The reason for the low prevalence of JIA-U in Japan may be related to the lower proportion of oligoarthritis in Japan than in North America and Europe. We compared the rate of uveitis in oligoarthritis patients and other arthritis patients between Japan and other countries (Table 6). The risk of uveitis in oligoarthritis patients in Japan is 10-fold, which is much higher than in oligoarthiritis patients in other countries. None of the patients with systemic arthritis and RF-positive polyarthritis developed uveitis - consistent with previous reports [2, 4, 5, 7, 18]. Because patients with these types of arthritis have little risk of developing uveitis, in such cases, autoinflammatory diseases such as Blau syndrome, or infections should be considered rather than JIA. In the present study, it was difficult to determine the frequency of the HLA-B27 allele because too few patients were tested, but Japanese JIA is characterized by a low incidence of enthesitis-related arthritis, as shown in Table 2. In addition, psoriatic arthritis is also very rare. Previous reports [19, 20] indicated that mean ANA titers tend to be high, with a significantly higher prevalence of ANA ≥1:320 in JIA patients with uveitis; our study also indicated a significant difference at ≥1:640.

The mean age of onset of JIA-U has been variably reported to be between 3.2–10.9 years [1, 2, 5, 18]; almost all cases are diagnosed before arthritis onset or within 4 years of onset [2, 4, 5, 15], and particularly within one year of the first ophthalmologic examination [2, 5] (not shown in Table 6). Furthermore, 10–24% patients develop uveitis before arthritis onset [1, 2, 18]. More than 80% of the locations affected by uveitis are anterior and 60.6 to 72% are bilateral [1, 2, 18]. Ocular complications including glaucoma, cataract, band-keratopathy, and posterior synechia at uveitis diagnosis were seen in between 37.3 and 56% of uveitis patients [1, 2]. Because the presence of complications will influence the visual prognosis, early diagnosis and treatment of uveitis is crucial. However, failure to have regular ocular examinations may lead to delayed diagnosis because JIA-U is asymptomatic and insidious in many cases [2]. In the present study, 36.6% of patients had ocular symptom at uveitis diagnosis; one of the reasons for the large number of patients with ocular symptoms may be the lack of regular routine ophthalmologic examinations in Japan. Similar to previous reports, the most commonly affected location of the uveitis was anterior, whereas fewer cases were bilateral. In our study, the median age at diagnosis of uveitis was 5 years, 80.5% of the patients developed uveitis before they reached 8 years of age, and 95.1% developed it under 7 years of arthritis onset. Thus, the risk of onset of uveitis is high for patients under 8 years of age and under 7 years from arthritis onset. These results suggest that recommendations for standard ophthalmological follow-up of Japanese JIA patients should consider patient age, and time from arthritis onset.

Except for two patients, all were treated with MTX, a medication previously shown to be effective for uveitis [21–23]. However, we could not determine whether treatment with MTX was effective in the present study.

Heiligenhaus et al. stated that ocular complications at the first visit to an ophthalmologist could be predictive of ocular complications at final observation [2]. In addition, Woreta et al. concluded that a short interval between arthritis and uveitis onset, ANA-positivity and the degree of ocular inflammation at the initial diagnosis, were all risk factors for complications [24]. Previous reports showed that cataract is associated with systemic steroid therapy, the amount of steroid eye drops and degree of ocular inflammation [25–28]. We found that the proportion of patients with complications increased from 31.7% at the first visit to an ophthalmologist to 56.1% at the final observation, and it was especially striking that the number of patients with cataract increased 3-fold over this period. Patients with complications at the initial diagnosis indeed had significantly more complications at the final observation, but there was no association between the presence of complications and age at diagnosis of uveitis, or the time from arthritis onset to uveitis diagnosis, and ANA-positivity. There were also no significant differences between complications and persisting active uveitis at the final observation. In addition, we found that systemic steroid therapy had no influence on cataract formation, and the presence of cataract at the first visit had no influence on the surgical history. JIA-U carries a risk of decreased visual acuity and blindness [29, 30]. While none of the patients lost their eyesight over the observation period in our study, more than half did not fully recover visual acuity or had decreased visual acuity at the final observation. Steroid eye drops, oral MTX, and biological agents (IFX and ADA) were mainly used for treatment, and especially biological agents had been employed in a high proportion of patients. This may be one of the reasons why there was no loss of eyesight and few patients with severely decreased vision in our study.

While female sex is reported as a risk factor for JIA-U, severity of the uveitis may be greater in males [31–33]. In comparison, in our study, female sex was not a risk factor for JIA-U, but males with uveitis did have a significantly higher incidence of active uveitis at the last observation.

This study has some limitations. It included 726 Japanese JIA patients, representing approximately 1/4 of all JIA patients in Japan and an estimated half of all patients treated in medical centers specialized in pediatric rheumatic diseases. Although Japan is divided into 47 prefectures, there are only about 80 pediatric rheumatologists and they are unevenly distribution locally, so general pediatricians treat JIA in areas where there are no pediatric rheumatologists. On the other hand, because severe cases are often referred to a pediatric rheumatologist, many JIA-U will be treated by pediatric rheumatologists. Accordingly, the prevalence of uveitis in Japan as established here may actually be even less than 6% of all JIA patients.

Just as there are few pediatric rheumatologists, so there are also few ophthalmologists who specialize in uveitis in Japan. In addition, although the standardization of uveitis nomenclature (SUN) working group reported criteria to evaluate uveitis [34], few ophthalmologists in Japan use these criteria. Therefore, in the present study, because pediatricians extracted the records written by each ophthalmologist there were some uninterpretable data, especially the location of uveitis. Because of the retrospective nature of this study, there were also some missing data such as data on visual acuity. Although we issued recommendations for ophthalmologic screening intervals for JIA in 2015 [9], because we issued it one year before this study, most patients were not receiving screening according to this recommendation. Hence, only 34% of patients had regular ophthalmologic screening before uveitis diagnosis, and 34% had ocular symptoms at the time of uveitis diagnosis in this study. JIA-U is typically asymptomatic and insidious [2], so if patients have regular ophthalmologic examinations, ocular symptoms may be identified less frequently at the time of JIA diagnosis, but more often beforehand.

We did not collect data on the degree of ocular inflammation and the dose of steroids, and so we were unable to analyze potential associations between uveitis and these factors. Also, we did not collect detailed data on dose and timing of drug use for any drugs other than steroids, so we could not analyze the effects of these treatments on uveitis. Thus, because detailed analysis of treatment was difficult in this study, we plan an additional study in cooperation with ophthalmologists in future.

## Conclusions

We investigated whether the prevalence and characteristics of JIA-U in Japan are different from those reported elsewhere. We conclude that the prevalence of JIA-U in Japan is lower than reported in countries with predominantly Caucasian populations. Risk factors for JIA-U were identified as oligoarthritis, early arthritis onset, ANA-positivity, RF-negativity and anti-CCP antibody-negativity. Arthritis onset was significantly earlier in females. Although the most frequent location of uveitis was anterior, males tended to have more affected locations in other the parts of the eye, and the uveitis remission rate was lower than in females. Thus, in males, uveitis may be more severe and harder to cure. Once uveitis had developed, many patients did not fully recover their eyesight. In addition, we found that uveitis may develop before arthritis onset, or under treatment for arthritis and even after cessation of arthritis medication. It is important to recognize these characteristics when caring for Japanese patients with JIA. Patients under 8 years of age and under 7 years of arthritis onset require especially careful specialist ophthalmological monitoring.

## Abbreviations

ADA: Adalimumab
ANA: Antinuclear antibodies
anti-CCP: anti-Cyclic citrullinated peptides
CI: Confidence interval
GC: Glucocorticoid
HLA-B27: Human leucocyte antigen B27
IFX: Infliximab
ILAR: International League of Associations for Rheumatology
JIA: Juvenile idiopathic arthritis
JIA-U: Juvenile idiopathic arthritis associated uveitis
MMP-3: Matrix Metalloproteinase-3
MTX: Methotrexate
NSAID: Non-steroidal anti-Inflammatory drugs
OR: Odds ratio
PRAJ: Pediatric Rheumatology Association of Japan
SUN: Standardization of Uveitis Nomenclature
TCZ: Tocilizumab
